# Carotid Artery Stiffness: Imaging Techniques and Impact on Cerebrovascular Disease

**DOI:** 10.3389/fcvm.2022.852173

**Published:** 2022-03-15

**Authors:** Hediyeh Baradaran, Ajay Gupta

**Affiliations:** ^1^Department of Radiology, University of Utah, Salt Lake City, UT, United States; ^2^Department of Radiology, Weill Cornell Medicine, New York, NY, United States; ^3^Feil Family Brain and Mind Research Institute, Weill Cornell Medicine, New York, NY, United States

**Keywords:** carotid artery disease, cerebrovascular disorders, cognitive dysfunction, magnetic resonance imaging, stroke

## Abstract

Arterial stiffness is an important measure of vascular aging and atherosclerosis. Though it is measured in many well-known epidemiologic cohort studies, arterial stiffness is often overlooked in routine clinical practice for a number of reasons including difficulties in measurement, variations in definition, and uncertainties surrounding treatment. Central arterial stiffness, a surrogate for aortic stiffness, is the most commonly measured marker of arterial stiffness. In addition to central stiffness, there are also a number of ultrasound based techniques to measure local vascular stiffness, including carotid stiffness. There is evidence that both local carotid stiffness and central arterial stiffness measures are associated with multiple cerebrovascular processes, including stroke and cognitive dysfunction. Mechanistic explanations supporting this association include increased flow load experienced by the cerebral microvasculature leading to cerebral parenchymal damage. In this article, we review definitions of carotid artery stiffness measures and pathophysiologic mechanisms underpinning its association with plaque development and downstream cerebral pathology. We will review the evidence surrounding the association of carotid stiffness measures with downstream manifestations including stroke, cerebral small vessel disease detected on brain MR such as white matter hyperintensities and covert brain infarctions, brain atrophy, and cognitive dysfunction. With consistent definitions, measurement methods, and further scientific support, carotid stiffness may have potential as an imaging-based risk factor for stroke and cognitive decline.

## Introduction

Diseases of the carotid artery are a major cause of worldwide morbidity and mortality, with carotid atherosclerosis accounting for nearly 1 in 5 acute ischemic strokes (1). Though stroke risk from carotid disease is most directly caused by wall thickening and plaque formation, the stiffening of the arterial wall likely plays an important role in the deleterious effects of carotid vascular disease (2). Though not routinely measured in clinical practice, arterial stiffness may play a role in downstream cerebrovascular ischemia, including contributing to stroke, cognitive impairment, and overall mortality (3–5). The exact role of arterial stiffness in contributing to these diseases is unclear as arterial stiffness increases with age and is associated with many conditions which are also associated with increased cardiovascular risk, such as hypertension, diabetes mellitus, and hypercholesterolemia. In addition to its association with downstream cognitive deficits, arterial stiffening may also potentiate the development of plaque (6), and may therefore play an under recognized role in increasing stroke risk.

Though arterial stiffness is not commonly measured in routine clinical practice, it has been well-studied in part because of its inclusion in multiple large epidemiologic cohort studies (7–9). Most commonly, arterial stiffness is a general term used to describe central arterial stiffness calculated via non-invasive techniques, usually pulse wave velocity (10). There are a number of methods used to measure arterial stiffness and the rigidity of a vessel wall both locally and systemically. The use of multiple techniques in various vascular beds accounts for some of the confusion regarding measuring stiffness measures (11). One of the barriers to widespread clinical adoption of arterial stiffness measurement is the lack of specific and uniform definitions and methods of measurement.

In this article, we will review common methods for measuring arterial stiffness including definitions of the most common measures of stiffness. We will also review mechanisms by which arterial stiffness may contribute to cerebrovascular ischemia, cognitive function, and carotid plaque formation.

## Arterial Stiffness Definitions and Measurements

**Figure 1:**
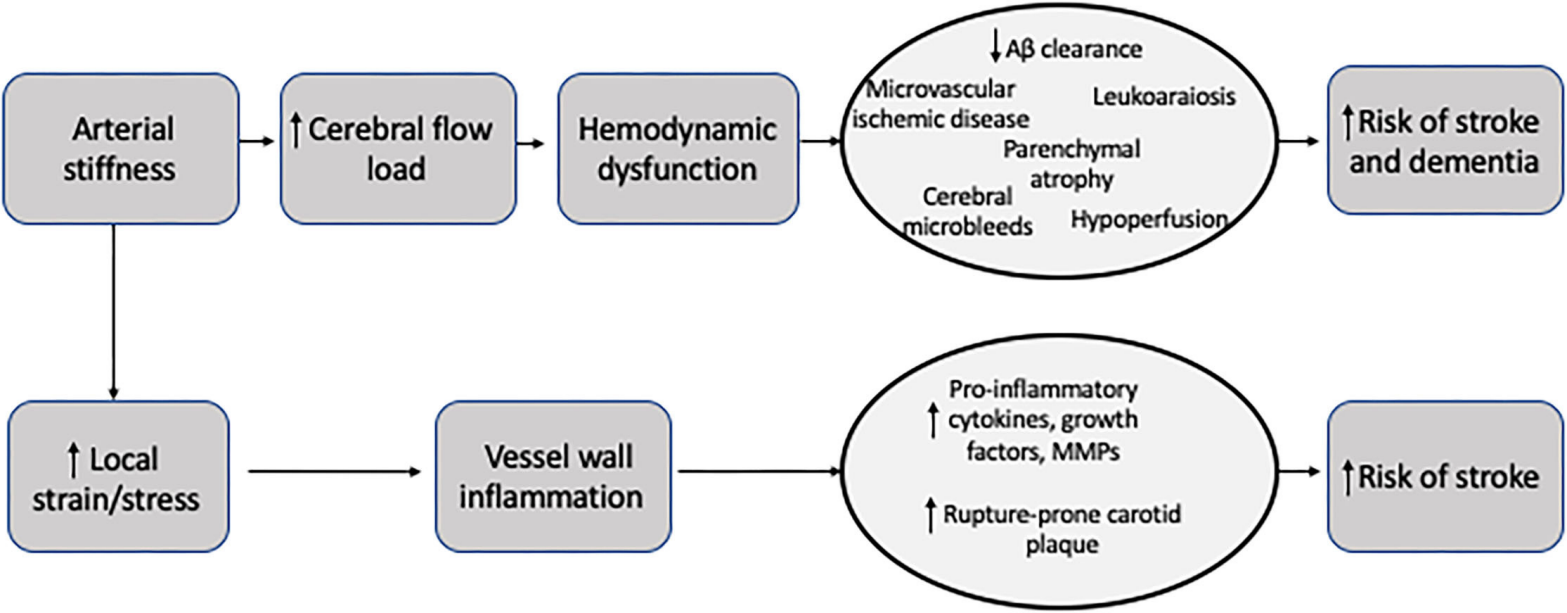
Schematic representation of changes to an artery wall in diastole compared to systole. The change in the diameter of the lumen is used in all local stiffness measurements.

Arterial stiffness is a general term to describe rigidity of the arterial walls. It is frequently assessed by measuring the velocity of a given pulse-wave in a specific vessel segment (12), though “stiffness” is also used more generally for direct measures of wall rigidity. There is further confusion surrounding the terminology because some stiffness measurements can be used in both local and systemic vascular beds and at times stiffness terms are used interchangeably. There is no single, unanimously agreed upon measurement to describe stiffness. Instead, there are several specific vascular wall properties or other pressure-related measurements which act as surrogates for stiffness. Further clouding the picture, the definition of arterial stiffness depends on whether local or central stiffness is being measured. We have searched the literature using PubMed for all articles referencing arterial and carotid stiffness published after 1990 in the English language. We considered studies including randomized controlled trials and retrospective and prospective cohort studies. We will briefly discuss the definitions of some of the most commonly acquired measures of arterial wall properties and their means of measurement (Table 1; Figure 1).

**Table 1:** Overview of arterial stiffness measurement techniques.

**Measure**	**Definition**	**Equation**	**Type of measure**	**Notes**
**Compliance**	For a given pressure change, the absolute change in arterial lumen area in systole	ΔD/ΔP	Local measure of stiffness	Higher compliance indicates more arterial stiffness
**Distensibility**	For a given pressure change, the relative change in arterial lumen area in systole	ΔD/ΔPxD	Local measure of stiffness	Accounts for arterial size
**Young's Elastic modulus**	The pressure change necessary for theoretical 100% stretch per unit area	ΔPxD/(ΔDxh)	Local measure of stiffness	Can account for wall thickness, such as intima-media thickness in the carotid artery
**Pulse wave velocity**	Velocity of travel of a pulse along a specific artery length	Distance/Δt	Central measure of stiffness	Most common measurement of arterial stiffness; surrogate for aortic stiffness

*P, pressure; D, diameter; h, wall thickness; t, time*.

### Pulse Wave Velocity

Pulse wave velocity (PWV) is perhaps the most commonly used and most well-known technique to evaluate arterial stiffness. PWV is a measure that evaluates the propagation of a wave through the vasculature by taking simultaneous recordings at two separate sites. The PWV increases as arterial stiffness increases. Carotid-femoral PWV (cfPWV) is often considered the gold standard of measuring central artery stiffness and is recommended by the American Heart Association with the highest level of supporting evidence as a method for evaluating arterial stiffness (13). This usually non-invasive method requires simultaneous measurement of pressure waves at two points across the body, in the case of cfPWV, the carotid and femoral arteries. The basic principal of PWV measurement involves recording pressure waves at two sites and then calculating transit time between those sites to determine PWV. This measurement can be accomplished with the help of a number of devices including applanation tonometers, echotracking devices, mechanotransducers, or Doppler probes (10).

One major limitation of cfPWV is that it is based on models for wave propagation through the arterial tree, which may not accurately reflect true stiffness, especially in aging arteries (10). cfPWV is a measure of central arterial stiffness and though the carotid artery is one of the main arteries measured, it is a surrogate measure for aortic stiffness, rather than a direct measure of carotid stiffness. Besides not being a direct measure of carotid stiffness, other limitations with using cfPWV include the difficulty in precisely measuring distances between arterial sites of measurement, especially in obese patients (10). Despite these limitations, cfPWV is the most commonly performed measure of arterial stiffness and has been routinely performed in many epidemiologic cohort studies. In fact, it serves as the primary method for measurement of many of the frequently cited studies describing arterial stiffness.

### Cardio-Ankle Vascular Index

Cardio-Ankle Vascular Index (CAVI) is another type of systemic measure of arterial stiffness which differs from PWV in that it is not dependent on blood pressure measurement (14). CAVI is a non-invasive method for measuring stiffness in the aorta, femoral, and tibial arteries and is strongly associated with the stiffness parameter ß (15). Further, there is evidence that CAVI is strongly associated with stroke and other cardiovascular events (16, 17). While not routinely used in clinical practice, CAVI is another non-invasive measure of arterial stiffness which may provide valuable information regarding cardiovascular risk.

### Local Stiffness Measures

Though PWV is the most commonly performed measure of central arterial stiffness, there are several measures for direct, local measurement of stiffness. Most of these measures of local arterial stiffness are determined via ultrasound techniques. Ultrasound is frequently performed throughout the cardiac cycle to determine changes in vessel diameter in diastole and systole. In addition to measuring luminal diameter to determine changes in volume throughout the cardiac cycle, ultrasound can also be used to measure wall thickness which can be used to determine the elastic properties of the arterial wall. Since most local stiffness measurement calculations require pressure measurements, local blood pressure measurements obtained via applanation tonometers are often calibrated to brachial pressures measured with standard sphygmomanometer. Given the lack of precise measurements on ultrasound, some studies utilize specific echotracking techniques to measure the lumen diameters which can provide more detailed measurements than ultrasound techniques (10). Local measurements of arterial stiffness can more directly measure arterial stiffness, rather than PWV which relies on assumptions from circulatory models and does not provide site-specific stiffness information. We will review several of the most common measures of local stiffness.

### Compliance

Compliance is a local measure of stiffness and can be used to directly measure carotid arterial wall stiffness. Compliance is defined as the ratio of any volume change (i.e., arterial lumen area) for a given pressure change. (ΔD/ ΔP cm/mm Hg). Compliance is an inverse measure of stiffness, meaning the greater the compliance, the lower the arterial stiffness. Larger arteries with higher cross-sectional volumes can general accommodate more volume for a given pressure change compared to smaller arteries.

### Distensibility

Distensibility is another local measure of stiffness. It is similar to compliance but can be more useful when comparing arteries of different sizes across the arterial tree. It is defined as the relative change in arterial lumen diameter or area for given pressure change [ΔD/ (ΔP x ΔD)].

### Elastic Modulus

Elastic modulus is another measure of stiffness that is defined as the pressure change necessary for theoretical 100% stretch (ΔP x ΔD)/ (ΔD). Young's elastic modulus is more commonly used and is defined as elastic modulus per unit area. Young's elastic modulus is used to measure elastic properties of a given vessel and can take the arterial wall thickness into account, including intima-media thickness in the carotid artery. Accounting for wall thickness is important, especially in the setting of atherosclerosis and plaque.

One of the reasons that arterial stiffness measures are not commonly performed in clinical practice is the difficulty in identifying the ideal measure for stiffness. While PWV is currently the simplest non-invasive method for measuring arterial stiffness, it has several limitations as discussed above, including inability to provide local stiffness measurement. In order for measures of arterial stiffness to become more mainstream, more uniformity in measurement techniques, definitions, and clinical application are necessary.

## Mechanisms By Which Arterial Stiffening Impacts the Brain

Arterial stiffness is a prominent feature of aging, but also a result of many systemic processes including atherosclerosis, diabetes, and even chronic renal disease. Stiffening is the product of complex interactions on the vessel wall, including hemodynamic forces, extrinsic effects such as hormonal changes and salt intake, and structural changes to the wall itself (18). Variations in the amount of collagen and elastin proteins within the arterial wall that occurs with aging can lead to structural alterations in the integrity of all layers of the vessel wall. In addition, smooth muscle tone changes, mediated by multiple factors including angiotensin II and nitric oxide, also result in changes to vascular compliance through changes in the media (18). Endothelial dysfunction may also play a role in the development of arterial stiffness via local reactive oxygen species. These complex diverse processes occur as a result of natural aging and are likely hastened by cardiovascular risk factors.

**Figure 2:**
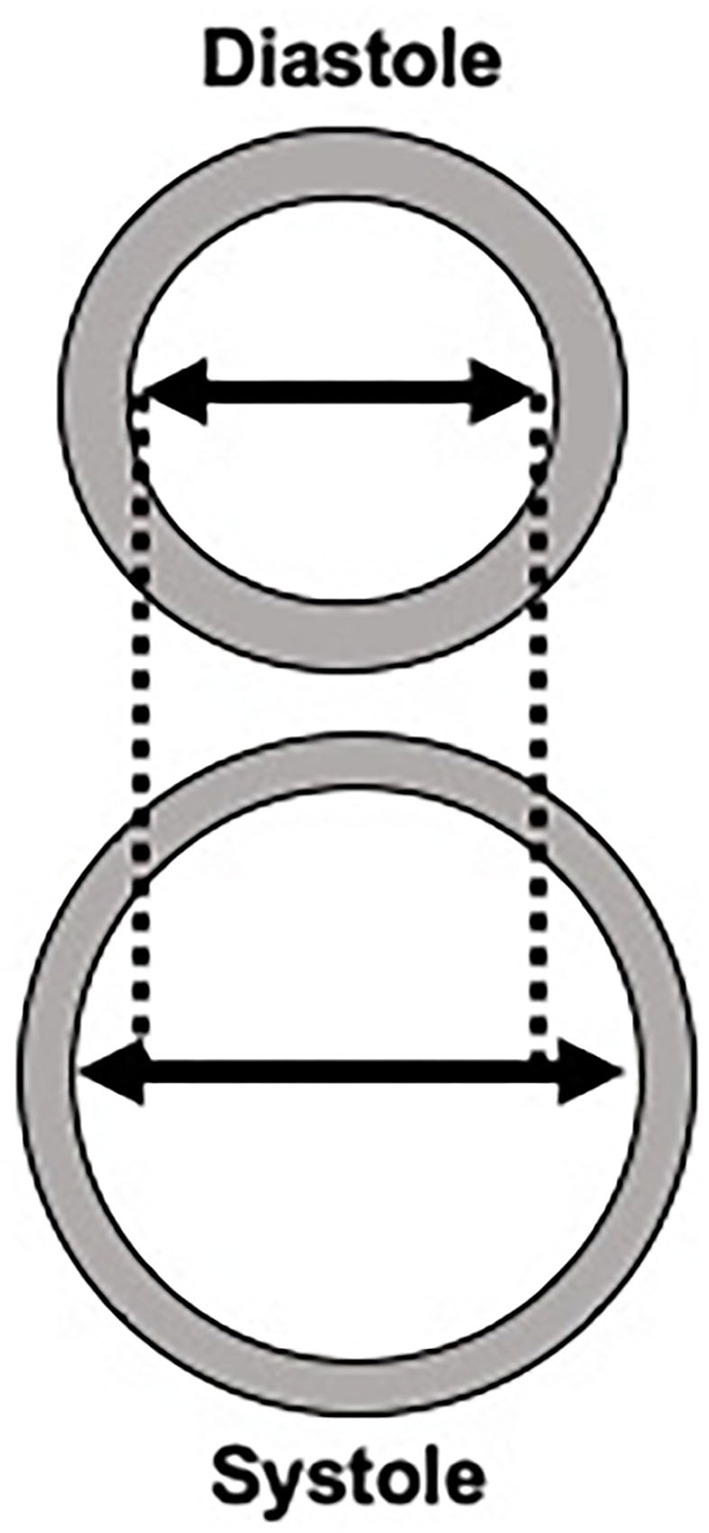
Potential mechanisms for the role of arterial stiffness in the development of stroke and dementia.

The exact role that carotid stiffness plays in the development of cerebrovascular ischemia and atherosclerosis is still under investigation. Given the shared risk factors for arterial stiffness and stroke, it is difficult to disentangle the complex relationship between stiffness, cardiovascular risk factors, and stroke. Still, one widely proposed link between arterial stiffness and cerebrovascular ischemia may be that arterial stiffness causes large arteries to become less responsive to normal pulsatile flow. This inability to respond to fluctuations of blood pressure throughout the cardiac cycle may result in more continuous perfusion, rather than pulsatile perfusion. Ultimately, more continuous perfusion results in increased flow load experienced by end-organs, especially the cerebral parenchyma (19, 20). The brain parenchyma is particularly susceptible to the increased flow load due to its overall low impedance leading to deep penetration of the flow load into the cerebral microvasculature (21). These alterations in the cerebral microvasculature appear to lead to parenchymal damage including ischemia and hemorrhage and other markers of small vessel disease. Eventually, this end-organ damage may manifest in a number of ways including stroke, cognitive dysfunction, or cerebral small vessel disease evident on brain imaging (Figure 2) (22).

Another potential mechanism by which arterial stiffness leads to stroke is through the development of plaque formation, specifically the formation of vulnerable plaque features (6). Emerging evidence suggests that arteries with increased stiffness may lead to increased shear strain in the adventitial layer. This increased strain may precipitate a local inflammatory reaction that facilitates plaque formation. Specifically, certain local stress signals and inflammatory responses, such as radical oxidants, growth factors and other pro-inflammatory cytokines can contribute to local endothelial dysfunction leading to increased angiogenesis and atherosclerotic plaque formation (18). Other studies have shown that increased arterial stiffness as measured by PWV is associated with increased calcified plaque (23). Further, there is a complex association between central arterial stiffness and aortic plaques with evidence that aortic plaques are also associated with downstream effects, including decreased brain volume and brain infarctions (24, 25). The exact relationship between arterial stiffness and the development of atherosclerotic plaque is a topic of considerable interest given that a more complete understanding of plaque formation may open new avenues for treating carotid atherosclerosis. This complex association may be more fully elucidated with the help of newer imaging technologies, such as optical coherence tomography, which allows for in vivo characterization of plaque as well as collagen and smooth muscle cells (26–29). This technology and others may help in furthering our mechanistic understanding of arterial stiffness.

Similarly, arterial stiffness may contribute to cerebral small vessel disease. Given the increased flow load experienced by the cerebral microvasculature in the setting of increased arterial stiffness, there is evidence of cerebral microvascular dysfunction and damage which can be detected on brain magnetic resonance (MR) imaging. The sequelae of cerebral microvascular damage can be seen as white matter hyperintensities, covert brain infarctions and cerebral microbleeds on MR. In addition, the increased flow load from altered cerebral microvascular flow leading to microstructural damage is thought to contribute to brain parenchymal atrophy, which is considered another marker of cerebral small vessel disease (30).

In addition to overt ischemia such as stroke, there is also evidence of downstream cognitive dysfunction in those with increased arterial stiffness. The microvascular damage from constant high flow loads leading to cerebral small vessel disease may in turn lead to cognitive impairment and dementia, given the very strong association between markers of cerebral small vessel disease and cognitive dysfunction (31–34). In addition to worsening cerebral small vessel disease, arterial stiffness may contribute to cognitive dysfunction by impaired blood flow leading to cerebral hypoperfusion, which may promote amyloid ß deposition (35). Another possibility is that arterial stiffness may impair amyloid clearance via decreased perivascular drainage of amyloid ß, leading to increased amyloid deposition which is seen in Alzheimer's disease (36–38).

## Evidence Supporting a Shared Link Between Arterial Stiffness and Brain Disease

### Stiffness and Stroke

Arterial stiffness may lead to stroke both because of increased flow load on the cerebral vasculature and due to accelerated development of rupture-prone atherosclerotic plaque in the carotid artery. Meta-analyses and individual-participant level data from epidemiologic cohorts demonstrate strong evidence of an association between central arterial stiffness and incident stroke (5, 39), including a study showing an association between central arterial stiffness and stroke independent of aortic plaque (24). There have also been several studies evaluating the association between local measures of carotid stiffness and incident stroke. A systematic review and meta-analysis of 22,472 participants found that greater carotid stiffness is associated with incident stroke (HR = 1.18; 95% CI 1.05–1.33), even when adjusting for cardiovascular risk factors (40, 41). When evaluating individual patient data and adjusting for cfPWV, a measure of central arterial stiffness, carotid stiffness was still statistically significantly associated with stroke.

Another study using a large epidemiologic cohort found that the addition of carotid distensibility measures improved ischemic stroke risk prediction (42). Accounting for baseline measures of carotid stiffness improved risk prediction beyond standard clinical markers for stroke risk and Framingham stroke risk score factors (40, 42). These findings suggest that carotid stiffness may be used as a tool for stroke risk stratification and prediction. Further confirmatory studies of this association are important, as is exploration of preventative and treatment methods of mitigating carotid stiffness.

Arterial stiffness also has a complex association with atrial fibrillation, which is the most common chronic arrhythmia and a strong predictor of stroke and mortality (43, 44). Several studies have shown that having high arterial stiffness and high pulsatile load increase the likelihood of developing atrial fibrillation (45–47). Conversely, having atrial fibrillation may itself lead to hemodynamic alterations including altered flow load leading to downstream effects (48). The relationship between atrial fibrillation and arterial stiffness warrants further evaluation to characterize this complex association, especially because atrial fibrillation is a treatable cause of ischemic stroke.

### Stiffness and Brain volume

Generalized cerebral parenchymal volume loss and hippocampal volume loss are commonly encountered findings on routine brain imaging. In addition to being closely associated with advancing age, low cerebral parenchymal volume is also associated with Alzheimer's disease, stroke, and generalized cognitive dysfunction (49). Multiple studies have shown a strong association between low carotid distensibility (or high carotid stiffness) with baseline lower total brain and cortical gray matter volume (22, 50). In addition to being associated with baseline lower cerebral volumes, there is evidence that carotid stiffness is associated with future lower parahippocampal and hippocampal volumes. Though the cause for this association is unclear, it is possible that arterial stiffness leads to generalized cerebral hypoperfusion which then, in turn, leads to atrophy. Another potential explanation is that high pressure pulsatility from arterial stiffness is transmitted to the cerebral microvasculature leading to microvascular dysfunction and damage in the brain, micro- and macrostructural neuronal damage, and ultimately leading to lower brain volumes (22, 51).

There are also a number of studies correlating aortic and central arterial stiffness with decreased parenchymal brain volume (52–54) though it is unclear if these studies can be generalized to carotid artery stiffness. In one of these studies, greater central arterial stiffness (cfPWV) was associated with lower volumes in signature Alzheimer's disease regions (53). There are several studies in large epidemiologic cohorts which have demonstrated the association between measures of arterial stiffness, as measured by cfPWV, to low total brain and hippocampal volumes, indicating a potential association with cognitive dysfunction.

### Stiffness and Markers of Cerebral Small Vessel Disease

Several markers of cerebral small vessel disease evident on routine brain imaging, including white matter hyperintensities (WMH), covert brain infarctions (CBI), and cerebral microbleeds (CMBs) are associated with mortality, stroke, cognitive dysfunction, and dementia (31, 32, 34, 55, 56). There is mixed evidence of an association between carotid artery stiffness and increased burden of cerebral small vessel disease. In a large epidemiologic cohort, baseline carotid artery stiffness was found to be associated with WMH 20 years later (8). Another small study found that local carotid stiffness measures were associated with MRI markers of cerebral small vessel disease (57). Other studies have also shown that increased arterial stiffness is associated with CMBs (58). All of these studies point to a potential association of carotid artery stiffness to downstream markers of cerebral small vessel disease, however, other cohorts found no association between carotid stiffness measures and markers of cerebral small vessel disease (59).

Despite the uncertainty surrounding the contribution of local carotid stiffness to markers of small vessel disease, there is stronger evidence of central arterial stiffness being associated with markers of cerebral small vessel disease, including white matter hyperintensities (53, 54). There are several systematic reviews and meta-analyses confirming the association between arterial stiffness, measured using multiple methods but most commonly with cfPWV, with white matter hyperintensities, cerebral microbleeds, and cerebral infarctions (4, 9, 60). Another study found that central arterial stiffness (cfPWV) was associated with overall burden of cerebral small vessel disease including lacunar infarctions and enlarged perivascular spaces in the basal ganglia (61).

### Stiffness and Cognitive Dysfunction

Several studies show that markers of central arterial stiffness are independent predictors of cognitive dysfunction. A recent cohort study found that higher central arterial stiffness (cfPWV) was associated with cognitive dysfunction and that association was in part mediated by markers of cerebral small vessel disease on brain MR (59). Data from the Framingham Heart Study suggest that central arterial stiffness, as measured by cfPWV, is an independent predictor of incident mild cognitive impairment within the entire cohort and incident dementia in the non-diabetic participants (62). Another longitudinal study found that increased central arterial stiffness (cfPWV) was associated with more pronounced cognitive decline in a cohort of institutionalized patients (63). Other longitudinal studies evaluating aortic stiffness have also confirmed an association with cognitive decline (64, 65). Other smaller studies have shown a cross-sectional association of central arterial stiffness with cognitive dysfunction (66–69).

The data supporting a link between carotid stiffness is less clear than for central arterial stiffness. For example, several studies have shown that carotid stiffness via distensibility or compliance measures is associated with cognitive dysfunction (70–75). Other studies have found no association between measures of carotid stiffness and cognitive dysfunction (7, 76). For example, one of the largest and most recent studies found no association between carotid distensibility and cognitive dysfunction after adjusting for potential confounders (59). The question of the association between direct measures of carotid stiffness and cognitive dysfunction is not definitively answered and future longitudinal studies would be helpful in elucidating this association.

One of the possible explanations for the association between central arterial stiffness and cognitive dysfunction, specifically Alzheimer's dementia is the thought that there is decreased clearance of amyloid protein, leading to disproportionate accumulation and the development of Alzheimer's dementia (36). Those individuals with higher central arterial stiffness (cfPWV) have been found to have worse performance of cognitive testing (53). Arterial stiffness as measured centrally with hcPWV (heart-carotid) was shown to be associated with lower brain volumes and higher white matter hyperintensity burden, but also greater amyloid-β deposition with an OR of 1.31 (95% CI 1.01–1.71) (54). One study following a large epidemiologic cohort, the Rotterdam study, found an association with PWV and poorer performance on the Stroop test but not on other cognitive tests (7). The study found no association between either central arterial stiffness (cfPWV) and carotid distensibility and cognitive dysfunction and dementia (7). Another study has shown that carotid stiffness specifically, rather than aortic stiffness, was independently associated with amyloid-β burden in patients with amnestic mild cognitive impairment after adjusting for confounders (77).

## Conclusion

Arterial stiffness, a specific measure of wall rigidity, can be measured both centrally in the aorta and locally in certain vascular beds such as the carotid artery. Having increased carotid stiffness is associated with multiple downstream effects including cardiovascular disease, stroke, and cognitive dysfunction. One of the leading pathophysiologic mechanisms underpinning these associations is the loss of vascular regulation from stiffness leading to increased flow load experienced by the cerebral parenchymal microvasculature. Both local measurements of carotid stiffness and measures of central arterial stiffness are variably associated with stroke, cognitive dysfunction, markers of cerebral small vessel disease, and low cerebral parenchymal volume. Currently, the lack of validated measurements precludes the routine use of measures of arterial stiffness in clinical practice. Future studies including those using optical With more standardized methods of measurements and continued validation, carotid stiffness measures may achieve greater clinical adoption as imaging-based risk factors for the development of stroke and dementia.

## Author Contributions

HB and AG designed the overall manuscript with the collection and assembly of data by HB. HB drafted manuscript with critical revision and feedback from AG. Both AG and HB gave final approval of the manuscript. All authors contributed to the article and approved the submitted version.

## Funding

HB was supported by a grant from the Association of University Radiologists- GE Radiology Research Academic Fellowship Award. AG was in part supported by National Institutes of Health grants R01HL144541 and R21HL145427. Research by HB was in part supported by the National Center for Advancing Translational Sciences of the National Institutes of Health under Award Number UL1TR002538.

## Conflict of Interest

The authors declare that the research was conducted in the absence of any commercial or financial relationships that could be construed as a potential conflict of interest.

## Publisher's Note

All claims expressed in this article are solely those of the authors and do not necessarily represent those of their affiliated organizations, or those of the publisher, the editors and the reviewers. Any product that may be evaluated in this article, or claim that may be made by its manufacturer, is not guaranteed or endorsed by the publisher.

## References

[1] Kolominsky-Rabas PL Weber M Gefeller O Neundoerfer B Heuschmann PU. Epidemiology of ischemic stroke subtypes according to TOAST criteria: incidence, recurrence, and long-term survival in ischemic stroke subtypes: a population-based study. Stroke. (2001) 32:2735–40. 10.1161/hs1201.100209 11739965

[2] O'Rourke MF Safar ME Dzau V. The Cardiovascular Continuum extended: aging effects on the aorta and microvasculature. Vasc Med. (2010) 15:461–8. 10.1177/1358863X10382946 21056945

[3] Mattace-Raso FU van der Cammen TJ Hofman A van Popele NM Bos ML Schalekamp M. Arterial stiffness and risk of coronary heart disease and stroke. Circulation. (2006) 113:657–63. 10.1161/CIRCULATIONAHA.105.555235 16461838

[4] Pase MP Herbert A Grima N Pipingas A O'Rourke MF. Arterial stiffness as a cause of cognitive decline and dementia: a systematic review and meta-analysis. Intern Med J. (2012) 42:808–15. 10.1111/j.1445-5994.2011.02645.x 22151013

[5] Vlachopoulos C Aznaouridis K Stefanadis C. Prediction of cardiovascular events and all-cause mortality with arterial stiffness: a systematic review and meta-analysis. J Am Coll Cardiol. (2010) 55:1318–27. 10.1016/j.jacc.2009.10.061 20338492

[6] Selwaness M. Bouwhuijsen Qvd Mattace-Raso FUS Verwoert GC Hofman A Franco OH. Arterial stiffness is associated with carotid intraplaque hemorrhage in the general population. Arterioscler Thromb Vasc Biol. (2014) 34:927–32. 10.1161/ATVBAHA.113.302603 24482373

[7] Poels MM van Oijen M Mattace-Raso FU Hofman A Koudstaal PJ Witteman JC. Arterial stiffness, cognitive decline, and risk of dementia: the Rotterdam study. Stroke. (2007) 38:888–92. 10.1161/01.STR.0000257998.33768.87 17272780

[8] de Havenon A Wong K-H Elkhetali A McNally J Majersik J Rost N. Carotid artery stiffness accurately predicts white matter hyperintensity volume 20 years later: a secondary analysis of the atherosclerosis risk in the community study. Am J Neuroradiol. (2019) 40:1369–73. 10.3174/ajnr.A6115 31248859 PMC7048485

[9] van Sloten TT Protogerou AD Henry RM Schram MT Launer LJ Stehouwer CD. Association between arterial stiffness, cerebral small vessel disease and cognitive impairment: a systematic review and meta-analysis. Neurosci Biobehav Rev. (2015) 53:121–30. 10.1016/j.neubiorev.2015.03.011 25827412 PMC5314721

[10] Laurent S Cockcroft J Van Bortel L Boutouyrie P Giannattasio C Hayoz D. Expert consensus document on arterial stiffness: methodological issues and clinical applications. Eur Heart J. (2006) 27:2588–605. 10.1093/eurheartj/ehl254 17000623

[11] Mackenzie IS Wilkinson IB Cockcroft JR. Assessment of arterial stiffness in clinical practice. QJM: Intern J Med. (2002) 95:67–74. 10.1093/qjmed/95.2.67 11861952

[12] O'Rourke MF Staessen JA Vlachopoulos C Duprez D. Clinical applications of arterial stiffness; definitions and reference values. Am J Hypertens. (2002) 15:426–44. 10.1016/S0895-7061(01)02319-6 12022246

[13] Townsend RR Wilkinson IB Schiffrin EL Avolio AP Chirinos JA Cockcroft JR. Recommendations for improving and standardizing vascular research on arterial stiffness: a scientific statement from the American heart association. Hypertension. (2015) 66:698–722. 10.1161/HYP.0000000000000033 26160955 PMC4587661

[14] Shirai K Utino J Otsuka K Takata M A. novel blood pressure-independent arterial wall stiffness parameter; cardio-ankle vascular index (CAVI). J Atheroscler Thromb. (2006) 13:101–7. 10.5551/jat.13.101 16733298

[15] Saiki A Sato Y Watanabe R Watanabe Y Imamura H Yamaguchi T. The role of a novel arterial stiffness parameter, cardio-ankle vascular index (CAVI), as a surrogate marker for cardiovascular diseases. J Atheroscler Throm. (2015) 23:32797. 10.5551/jat.32797 26607350

[16] Sato Y Yoshihisa A Ichijo Y Watanabe K Hotsuki Y Kimishima Y. Cardio-ankle vascular index predicts post-discharge stroke in patients with heart failure. J Atheroscler Throm. (2020) 25:58727. 10.5551/jat.58727 32981919 PMC8265923

[17] Otsuka K Fukuda S Shimada K Suzuki K Nakanishi K Yoshiyama M. Serial assessment of arterial stiffness by cardio-ankle vascular index for prediction of future cardiovascular events in patients with coronary artery disease. Hypertension Research. (2014) 37:1014–20. 10.1038/hr.2014.116 25007768

[18] Zieman SJ Melenovsky V Kass DA. Mechanisms, pathophysiology, and therapy of arterial stiffness. Arterioscler Thromb Vasc Biol. (2005) 25:932–43. 10.1161/01.ATV.0000160548.78317.29 15731494

[19] Mitchell GF. Effects of central arterial aging on the structure and function of the peripheral vasculature: implications for end-organ damage. J Appl Physiol. (2008) 105:1652–60. 10.1152/japplphysiol.90549.2008 18772322 PMC2584844

[20] van Sloten TT Schram MT van den Hurk K Dekker JM Nijpels G Henry RM. Local stiffness of the carotid and femoral artery is associated with incident cardiovascular events and all-cause mortality: the Hoorn study. J Am Coll Cardiol. (2014) 63:1739–47. 10.1016/j.jacc.2013.12.041 24583306

[21] Climie RE van Sloten TT Bruno R-M Taddei S Empana J-P Stehouwer CD. Macrovasculature and microvasculature at the crossroads between type 2 diabetes mellitus and hypertension. Hypertension. (2019) 73:1138–49. 10.1161/HYPERTENSIONAHA.118.11769 31067192

[22] Mitchell GF van Buchem MA Sigurdsson S Gotal JD Jonsdottir MK Kjartansson Ó. Arterial stiffness, pressure and flow pulsatility and brain structure and function: the age, gene/environment susceptibility–Reykjavik study. Brain. (2011) 134:3398–407. 10.1093/brain/awr253 22075523 PMC3212721

[23] Cecelja M Jiang B Bevan L Frost ML Spector TD Chowienczyk PJ. Arterial stiffening relates to arterial calcification but not to non-calcified atheroma in women. J Am Coll Cardiol. (2011) 57:1480–6. 10.1016/j.jacc.2010.09.079 21435518 PMC3919172

[24] Sugioka K Hozumi T Sciacca RR Miyake Y Titova I Gaspard G. Impact of aortic stiffness on ischemic stroke in elderly patients. Stroke. (2002) 33:2077–81. 10.1161/01.STR.0000021410.83049.32 12154266

[25] Aparicio HJ Petrea RE Massaro JM Manning WJ Oyama-Manabe N Beiser AS. Association of descending thoracic aortic plaque with brain atrophy and white matter hyperintensities: the Framingham heart study. Atherosclerosis. (2017) 265:305–11. 10.1016/j.atherosclerosis.2017.06.919 28673480 PMC5617776

[26] Ughi GJ Marosfoi MG King RM Caroff J Peterson LM Duncan BH. A neurovascular high-frequency optical coherence tomography system enables *in situ* cerebrovascular volumetric microscopy. Nat Commun. (2020) 11:1–10. 10.1038/s41467-020-17702-7 32737314 PMC7395105

[27] Chen C-J Kumar JS Chen SH Ding D Buell TJ Sur S. Optical coherence tomography: future applications in cerebrovascular imaging. Stroke. (2018) 49:1044–50. 10.1161/STROKEAHA.117.019818 29491139

[28] Otsuka K Villiger M Nadkarni SK Bouma BE. Intravascular polarimetry: clinical translation and future applications of catheter-based polarization sensitive optical frequency domain imaging. Front Cardiovas Med. (2020) 7:146. 10.3389/fcvm.2020.00146 33005632 PMC7485575

[29] Otsuka K Villiger M Karanasos A van Zandvoort LJ Doradla P Ren J. Intravascular polarimetry in patients with coronary artery disease. Cardiovascular Imaging. (2020) 13:790–801. 10.1016/j.jcmg.2019.06.015 31422135 PMC7241775

[30] Wardlaw JM Smith EE Biessels GJ Cordonnier C Fazekas F Frayne R. Neuroimaging standards for research into small vessel disease and its contribution to ageing and neurodegeneration. Lancet Neurol. (2013) 12:822–38. 10.1016/S1474-4422(13)70124-8 23867200 PMC3714437

[31] Vermeer SE Prins ND den Heijer T Hofman A Koudstaal PJ Breteler MM. Silent brain infarcts and the risk of dementia and cognitive decline. N Eng J Med. (2003) 348:1215–22. 10.1056/NEJMoa022066 12660385

[32] Debette S Seshadri S Beiser A Au R Himali JJ Palumbo C. Midlife vascular risk factor exposure accelerates structural brain aging and cognitive decline. Neurology. (2011) 77:461–8. 10.1212/WNL.0b013e318227b227 21810696 PMC3146307

[33] Debette S Markus HS. The clinical importance of white matter hyperintensities on brain magnetic resonance imaging: systematic review and meta-analysis. BMJ. (2010) 341:666. 10.1136/bmj.c3666 20660506 PMC2910261

[34] Debette S Beiser A DeCarli C Au R Himali JJ Kelly-Hayes M. Association of MRI markers of vascular brain injury with incident stroke, mild cognitive impairment, dementia, and mortality. Stroke. (2010) 41:600–6. 10.1161/STROKEAHA.109.570044 20167919 PMC2847685

[35] Ruitenberg A den Heijer T Bakker SL van Swieten JC Koudstaal PJ Hofman A. Cerebral hypoperfusion and clinical onset of dementia: the Rotterdam study. Ann Neurol. (2005) 57:789–94. 10.1002/ana.20493 15929050

[36] Mawuenyega KG Sigurdson W Ovod V Munsell L Kasten T Morris JC. Decreased clearance of CNS β-amyloid in Alzheimer's disease. Science. (2010) 330:1774. 10.1126/science.1197623 21148344 PMC3073454

[37] de la Torre JC. Is Alzheimer's disease a neurodegenerative or a vascular disorder? Data, dogma, and dialectics. Lancet Neurol. (2004) 3:184–90. 10.1016/S1474-4422(04)00683-0 14980533

[38] Gupta A Iadecola C. Impaired Aβ clearance: a potential link between atherosclerosis and Alzheimer's disease. Front Aging Neurosci. (2015) 7:115. 10.3389/fnagi.2015.00115 26136682 PMC4468824

[39] Ben-Shlomo Y Spears M Boustred C May M Anderson SG Benjamin EJ. Aortic pulse wave velocity improves cardiovascular event prediction: an individual participant meta-analysis of prospective observational data from 17,635 subjects. J Am Coll Cardiol. (2014) 63:636–46. 10.1016/j.jacc.2013.09.063 24239664 PMC4401072

[40] Sloten TTv Sedaghat S Laurent S London GM Pannier B Ikram MA. Carotid stiffness is associated with incident stroke. J Am College Cardiol. (2015) 66:2116–25. 10.1016/j.jacc.2015.08.888 26541923

[41] Dijk JM. Graaf Yvd, Grobbee DE, Bots ML. Carotid stiffness indicates risk of ischemic stroke and TIA in patients with internal carotid artery stenosis. Stroke. (2004) 35:2258–62. 10.1161/01.STR.0000141702.26898.e9 15331793

[42] Baradaran H Delic A Wong KH Sheibani N Alexander M McNally JS. Using ultrasound and inflammation to improve prediction of ischemic stroke: a secondary analysis of the multi-ethnic study of atherosclerosis. Cerebrovasc Dis Extra. (2021) 11:37–43. 10.1159/000514373 33601394 PMC7989729

[43] Wolf PA Abbott RD Kannel WB. Atrial fibrillation as an independent risk factor for stroke: the Framingham study. stroke. (1991) 22:983–8. 10.1161/01.STR.22.8.983 1866765

[44] Benjamin EJ Wolf PA D'Agostino RB Silbershatz H Kannel WB Levy D. Impact of atrial fibrillation on the risk of death: the Framingham Heart Study. Circulation. (1998) 98:946–52. 10.1161/01.CIR.98.10.946 9737513

[45] Cremer A Lainé M Papaioannou G Yeim S Gosse P. Increased arterial stiffness is an independent predictor of atrial fibrillation in hypertensive patients. J Hypertens. (2015) 33:2150–5. 10.1097/HJH.0000000000000652 26431194

[46] Chen LY Foo DC Wong RC Seow S-C Gong L Benditt DG. Increased carotid intima-media thickness and arterial stiffness are associated with lone atrial fibrillation. Int J Cardiol. (2013) 168:3132–4. 10.1016/j.ijcard.2013.04.034 23623340

[47] Shaikh AY Wang N Yin X Larson MG Vasan RS Hamburg NM. Relations of arterial stiffness and brachial flow 2013; mediated dilation with new-onset atrial fibrillation. Hypertension. (2016) 68:590–6. 10.1161/HYPERTENSIONAHA.116.07650 27456517

[48] Saji N Toba K Sakurai T. Cerebral small vessel disease and arterial stiffness: tsunami effect in the brain. Pulse. (2015) 3:182–9. 10.1159/000443614 27195239 PMC4865071

[49] Chao L Mueller S Buckley S Peek K Raptentsetseng S Elman J. Evidence of neurodegeneration in brains of older adults who do not yet fulfill MCI criteria. Neurobiol Aging. (2010) 31:368–77. 10.1016/j.neurobiolaging.2008.05.004 18550226 PMC2814904

[50] Jochemsen HM Muller M Bots ML Scheltens P Vincken KL Mali WP. Arterial stiffness and progression of structural brain changes: the SMART-MR study. Neurology. (2015) 84:448–55. 10.1212/WNL.0000000000001201 25552578

[51] Mitchell GF Vita JA Larson MG Parise H Keyes MJ Warner E. Cross-sectional relations of peripheral microvascular function, cardiovascular disease risk factors, and aortic stiffness: the Framingham heart study. Circulation. (2005) 112:3722–8. 10.1161/CIRCULATIONAHA.105.551168 16330686

[52] Maillard P Mitchell GF Himali JJ Beiser A Tsao CW Pase MP. Effects of arterial stiffness on brain integrity in young adults from the framingham heart study. Stroke. (2016) 47:1030–6. 10.1161/STROKEAHA.116.012949 26965846 PMC4811686

[53] Palta P Sharrett AR Wei J Meyer ML Kucharska-Newton A Power MC. Central arterial stiffness is associated with structural brain damage and poorer cognitive performance: the ARIC study. J Am Heart Assoc. (2019) 8:e011045. 10.1161/JAHA.118.011045 30646799 PMC6497348

[54] Hughes TM Wagenknecht LE Craft S Mintz A Heiss G Palta P. Arterial stiffness and dementia pathology: atherosclerosis risk in communities (ARIC)-PET study. Neurology. (2018) 90:e1248–e56. 10.1212/WNL.0000000000005259 29549223 PMC5890613

[55] Vermeer SE Koudstaal PJ Oudkerk M Hofman A Breteler MM. Prevalence and risk factors of silent brain infarcts in the population-based Rotterdam scan study. Stroke. (2002) 33:21–5. 10.1161/hs0102.101629 11779883

[56] Bernick C Kuller L Dulberg C Longstreth Jr W Manolio T Beauchamp N. Silent MRI infarcts and the risk of future stroke The cardiovascular health study. Neurology. (2001) 57:1222–9. 10.1212/WNL.57.7.1222 11591840

[57] Huang X Kang X Xue J Kang C Lv H Li Z. Evaluation of carotid artery elasticity changes in patients with cerebral small vessel disease. Int J Clin Exp Med. (2015) 8:18825–30. 26770502 PMC4694402

[58] Ding J Mitchell GF Bots ML Sigurdsson S Harris TB Garcia M. Carotid arterial stiffness and risk of incident cerebral microbleeds in older people: the age, gene/environment susceptibility (AGES)-Reykjavik study. Arterioscler Thromb Vasc Biol. (2015) 35:1889–95. 10.1161/ATVBAHA.115.305451 26112009 PMC4514556

[59] Rensma SP Stehouwer CD Van Boxtel MP Houben AJ Berendschot TT Jansen JF. Associations of arterial stiffness with cognitive performance, and the role of microvascular dysfunction: the maastricht study. Hypertension. (2020) 75:1607–14. 10.1161/HYPERTENSIONAHA.119.14307 32275192

[60] Singer J Trollor JN Baune BT Sachdev PS Smith E. Arterial stiffness, the brain, and cognition: a systematic review. Ageing Res Rev. (2014) 15:16–27. 10.1016/j.arr.2014.02.002 24548924

[61] Riba-Llena I Jiménez-Balado J Castañé X Girona A López-Rueda A Mundet X. Arterial stiffness is associated with basal ganglia enlarged perivascular spaces and cerebral small vessel disease load. Stroke. (2018) 49:1279–81. 10.1161/STROKEAHA.118.020163 29669870

[62] Pase MP Beiser A Himali JJ Tsao C Satizabal CL Vasan RS. Aortic stiffness and the risk of incident mild cognitive impairment and dementia. Stroke. (2016) 47:2256–61. 10.1161/STROKEAHA.116.013508 27491735 PMC4995162

[63] Benetos A Watfa G Hanon O Salvi P Fantin F Toulza O. Pulse wave velocity is associated with 1-year cognitive decline in the elderly older than 80 years: the PARTAGE study. J Am Med Dir Assoc. (2012) 13:239–43. 10.1016/j.jamda.2010.08.014 21450208

[64] Watson NL Sutton-Tyrrell K Rosano C Boudreau RM Hardy SE Simonsick EM. Arterial stiffness and cognitive decline in well-functioning older adults. J Gerontol Series A: Biomed Sci Med Sci. (2011) 66:1336–42. 10.1093/gerona/glr119 21768503 PMC3210954

[65] Elias MF Robbins MA Budge MM Abhayaratna WP Dore GA Elias PK. Arterial pulse wave velocity and cognition with advancing age. Hypertension. (2009) 53:668–73. 10.1161/HYPERTENSIONAHA.108.126342 19237680 PMC2716128

[66] Muela HC Costa-Hong VA Yassuda MS Moraes NC Memória CM Machado MF. Higher arterial stiffness is associated with lower cognitive performance in patients with hypertension. J Clin Hyperten. (2018) 20:22–30. 10.1111/jch.13129 29106057 PMC8031309

[67] Mehrabian S Raycheva M Gateva A Todorova G Angelova P Traykova M. Cognitive dysfunction profile and arterial stiffness in type 2 diabetes. J Neurol Sci. (2012) 322:152–6. 10.1016/j.jns.2012.07.046 22871541

[68] Scuteri A Brancati AM Gianni W Assisi A Volpe M. Arterial stiffness is an independent risk factor for cognitive impairment in the elderly: a pilot study. J Hypertens. (2005) 23:1211–6. 10.1097/01.hjh.0000170384.38708.b7 15894897

[69] Triantafyllidi H Arvaniti C Lekakis J Ikonomidis I Siafakas N Tzortzis S. Cognitive impairment is related to increased arterial stiffness and microvascular damage in patients with never-treated essential hypertension. Am J Hypertens. (2009) 22:525–30. 10.1038/ajh.2009.35 19265790

[70] Huck DM Hanna DB Rubin LH Maki P Valcour V Springer G. Carotid artery stiffness and cognitive decline among women with or at risk for HIV infection. J Acq Immune Def Syn 1999. (2018) 78:338. 10.1097/QAI.0000000000001685 29578932 PMC5997527

[71] Geijselaers SL Sep SJ Schram MT van Boxtel MP van Sloten TT Henry RM. Carotid stiffness is associated with impairment of cognitive performance in individuals with and without type 2 diabetes. The Maastricht Study Atherosclerosis. (2016) 253:186–93. 10.1016/j.atherosclerosis.2016.07.912 27503567

[72] DuBose LE Voss MW Weng TB Kent JD Dubishar KM Lane-Cordova A. Carotid β-stiffness index is associated with slower processing speed but not working memory or white matter integrity in healthy middle-aged/older adults. J Appl Physiol. (2017) 122:868–76. 10.1152/japplphysiol.00769.2016 28126907 PMC5407200

[73] Lim SL Gao Q Nyunt MSZ Gong L Lunaria JB Lim ML. Vascular health indices and cognitive domain function: singapore longitudinal ageing studies. J Alzheimers Dis. (2016) 50:27–40. 10.3233/JAD-150516 26639958

[74] TarumiTGonzalesMMFallowBNualnimNPyronMTanakaH. Central artery stiffness, neuropsychological function, and cerebral perfusion in sedentary and endurance-trained middle-aged adults. J Hypertens. (2013) 31:2400–9. 10.1097/HJH.0b013e328364decc24220591

[75] Hoth KF Moreau KL Weinberger HD Holm KE Meschede K Crapo JD. Carotid artery stiffness is associated with cognitive performance in former smokers with and without chronic obstructive pulmonary disease. J Am Heart Assoc. (2020) 9:e014862. 10.1161/JAHA.119.014862 32338117 PMC7428572

[76] Chiesa ST Masi S Shipley MJ Ellins EA Fraser AG Hughes AD. Carotid artery wave intensity in mid-to late-life predicts cognitive decline: the Whitehall II study. Eur Heart J. (2019) 40:2300–9. 10.1093/eurheartj/ehz189 30957863 PMC6642727

[77] Pasha EP Rutjes E Tomoto T Tarumi T Stowe A Claassen JA. Carotid stiffness is associated with brain amyloid-β burden in amnestic mild cognitive impairment. J Alzheimers Dis. (2020) 74:1–11. 10.3233/JAD-191073 32083583

